# SeqEntropy: Genome-Wide Assessment of Repeats for Short Read Sequencing

**DOI:** 10.1371/journal.pone.0059484

**Published:** 2013-03-27

**Authors:** Hsueh-Ting Chu, William WL. Hsiao, Theresa TH. Tsao, D. Frank Hsu, Chaur-Chin Chen, Sheng-An Lee, Cheng-Yan Kao

**Affiliations:** 1 Department of Biomedical informatics, Asia University, Taichung, Taiwan; 2 Department of Computer Science and Information Engineering, Asia University, Taichung, Taiwan; 3 British Columbia Public Health Microbiology and Reference Laboratory, Vancouver, British Columbia, Canada; 4 Department of Pathology and Laboratory Medicine, University of British Columbia, Vancouver, British Columbia, Canada; 5 Department of Computer Science and Information Engineering, National Taiwan University, Taipei, Taiwan; 6 Department of Computer and Information Science, Fordham University, New York, New York, United States of America; 7 Department of Computer Science, National Tsing Hua University, Hsinchu, Taiwan; 8 Department of Information Management, Kainan University, Taoyuan, Taiwan; Albert Einstein College of Medicine, United States of America

## Abstract

**Background:**

Recent studies on genome assembly from short-read sequencing data reported the limitation of this technology to reconstruct the entire genome even at very high depth coverage. We investigated the limitation from the perspective of information theory to evaluate the effect of repeats on short-read genome assembly using idealized (error-free) reads at different lengths.

**Methodology/Principal Findings:**

We define a metric H^(k)^ to be the entropy of sequencing reads at a read length k and use the relative loss of entropy ΔH^(k)^ to measure the impact of repeats for the reconstruction of whole-genome from sequences of length k. In our experiments, we found that entropy loss correlates well with de-novo assembly coverage of a genome, and a score of ΔH^(k)^>1% indicates a severe loss in genome reconstruction fidelity. The minimal read lengths to achieve ΔH^(k)^<1% are different for various organisms and are independent of the genome size. For example, in order to meet the threshold of ΔH^(k)^<1%, a read length of 60 bp is needed for the sequencing of human genome (3.2 10^9^ bp) and 320 bp for the sequencing of fruit fly (1.8×10^8^ bp). We also calculated the ΔH^(k)^ scores for 2725 prokaryotic chromosomes and plasmids at several read lengths. Our results indicate that the levels of repeats in different genomes are diverse and the entropy of sequencing reads provides a measurement for the repeat structures.

**Conclusions/Significance:**

The proposed entropy-based measurement, which can be calculated in seconds to minutes in most cases, provides a rapid quantitative evaluation on the limitation of idealized short-read genome sequencing. Moreover, the calculation can be parallelized to scale up to large euakryotic genomes. This approach may be useful to tune the sequencing parameters to achieve better genome assemblies when a closely related genome is already available.

## Introduction

The development of the next generation sequencing technologies (NGS) raised the hope to conduct true haplotype analysis of human genome [1] and for rapid full genome sequencing and typing of various organisms. The 1000 Genomes Project, launched in 2008, began to sequence one thousand human genomes with SGS platforms [2]. In the first phase of the project, the goal was to generate low coverage whole genome shotgun sequencing of 185 individuals. These data were produced in order to validate millions of published genetic variations including single nucleotide polymorphisms (SNPs), insertions and deletions (indels), and other structural variants. Soon after the announcement of the project, another group of scientists started the Genome 10 K project in 2009 which aims to “assemble a genomic zoo” by sequencing the genomes of vertebrate animals [3]. These studies help us understand the correlation between genotypes and phenotypes if large-scale genome shotgun sequencing could be unambiguously and accurately assembled.

Recently, Alkan *et al.* published their analysis of the short-read sequencing data generated from the whole genomes of a Han Chinese individual and a Yoruban individual [4]. In contrary to the initial optimistic view of using NGS technologies to reconstitute the whole genome, it showed a severe deficiency in disambiguating certain genomic regions with short reads. Compared to reference human genome, more than 400 mega-base-pairs (Mbps) of common repeats are missing. As a consequence, it is still a challenge to perform accurate haplotype analysis even though a massive amount of genome sequencing data from multiple individuals is currently available. In another study by Kingsford *et al.*, they explored the effect of repeats in prokaryotic genome assembly using de Bruijn graphs and derived an upper bound of contig sizes for a large number of prokaryotic genomes based on simulated short-reads of different lengths [5]. They concluded that while most genes (>98%) can be recovered in contigs derived from reads as short as 100 bps, even reads as long as 1000 bps are not sufficient to produce a complete prokaryotic genome in most cases.

In this paper, we investigated the impact of read length with a different quantitative analysis. We define the entropy of nucleotide fragments (H) and use the loss in entropy to measure the influence of repeats on genome assembly. The repeat problem has plagued the assembly process since the first generation of sequencers [6], [7]. Regardless of the sequence assembler used, the de novo assembly of sequencing data will collapse identical repeats if the length of repeated segments is greater than the read length, resulting in incomplete genome reconstruction. As a result, with the read length limitations imposed by sequencing platforms, repetitive regions will not be reconstructed. In information theory, the entropy score is an index to measure the disorder in a system. Thus we use the definition of k-substring entropy to represent the expected value of the quantified information contained in the reads of length k produced by the sequencing procedure. We apply this measurement to both prokaryotic and eukaryotic genomes, including the human genome. We demonstrated the usefulness of the score as a measurement of the repeat structures of the genomes and proposed how it can be used to aid genome sequencing efforts from the perspective of read lengths and repetitiveness of target genomes.

## Materials and Methods

### An Idealized Model of Short Read Sequencing

**Figure 1:**
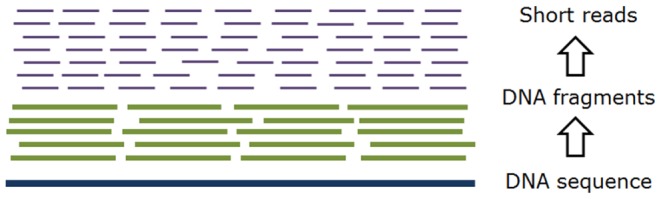
Model of typical short read sequencing. (a) The target sequence is randomly broken into fragments and filtered by their lengths to form a sequencing library. (b) The end or ends of the DNA fragments are sequenced in parallel to generate a massive set of short reads. We assumed the sequencing is random so that each position is more or less covered by equal numbers of fixed-length reads.

In Figure 1, we illustrated a simplified model of the process to generate high coverage sequence data using a modern sequencing platform such as the Illumina/Solexa system. We considered fixed read length systems for this study since such systems currently provide the cost-effectiveness required for large-scale sequencing and have wider market adoptions. For our current purpose, we simplified the sequencing into two major steps. In the first step, a target DNA sequence is broken into smaller fragments. The fragments are filtered by size and then form a sequencing library. The second step is the parallel sequencing of the ends of these fragments. Current parallelized sequencing technologies are based on various sequencing-by-synthesis methods which can produce a massive number of reads with high redundancy. In this model, we assume that both of the steps are random. That is, the produced sequencing reads can be from any position in the DNA sequence with equal probability although various factors can contribute to sequence sampling bias in real life - resulting in uneven coverage and gaps in sequence assembly [8]. In addition, the reads can come from both the forward strand T_F_ and the reverse strand T_R_ of the target DNA sequence T. For convenience, we just use T to present both strands T_F_ and T_R_ in the following sections.

### The Computation of k-substring Entropy Loss as a Quantitative Measurement of Repeats

If we can filter out all sequencing errors, we can see in Figure 1 that the reads generated are substrings of the target DNA sequence. Let *x* be a substring of length *k* from DNA sequence T = t_1_t_2_t_3_…t_m_ where each t_j_, 1≤ j ≤ m, is one of the nucleotides {A, C, G, T}.

In other words, for short read sequencing with reads of length *k*, the reads could be denoted as
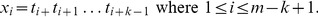
(1)


Let *S_k_* be the collection of all possible substrings *x_i_* = *t_i+1_ t_i+2_* …*t_i+k_* with length *k* where each t_j_∈{A, C, G, T}.

Define
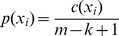
(2)where c(*x_i_*) is the number of occurrences of *x_i_* in the sequence T.

The Shannon entropy H of S is defined as
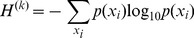
(3)where *k* is the length of substrings [9].

In particular, if any substring x_i_ = *t_i+1_ t_i+2_* …*t_i+k_* of length *k* is unique. Then,
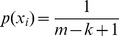
(4)


If any substring of length *k* is unique, there is no repeat whose length is greater than or equal to *k*. In this case, it achieves a maximum of entropy
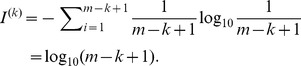
(5)


In almost all of the applications, we have
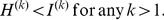
(6)


We define the relative entropy loss of length *k* as
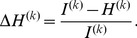
(7)


If there are a large number of substrings {*x_i_*}, we can divide the substrings into independent sets *T_1_*, *T_2_*, ..., *T_m_* according their prefixes. Then we can rewrite (3) as
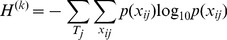
(8)


Equation 8 represents an approach to divide up the input into smaller sets and to process them in parallel. This tactic allows one to process a large genome in a timely manner using modest computing resources.

## Results

### Deficiency in Sequence Coverage Caused by Sequence Repeats is Strongly Correlated to Loss in k-substring Entropy

We investigated the impact of repeat structures on genome assembly and the correlation of sequence coverage and k-substring entropy using the SeqEntropy program that we developed by comparing the sequence coverage results obtained from the SHARCGS de-novo assembler paper [10] and our entropy measurements. Table 1 lists the BAC insert sequences derived from *Arabidopsis thaliana* and *Drosophila melanogaster* (fruit fly). The reconstructed sequence coverage of assembly was quoted from the original SHARCGS paper. The result showed a strong correlation between the ratio of reconstructed coverage and entropy loss. Most BAC sequences have small deficiency from the reconstructed sequence coverage except the following three sequences: AC009243, AC092242 and AC007329 (the bolded rows in Table 1). These three sequences have significantly incomplete coverage such that the sequence coverage by SHARCGS assembled contigs is less than 90%. All of these three sequences lose more than 1% entropy at read length of 30 bp. Consequently, we propose that >1% entropy loss results in poor assembly results using de novo assemblers. For comparison, we also calculated the percentage of simple repeats in these BACs and showed the results in the last column of Table 1. The proposed relative entropy loss scores correlate well with the percentages of repeats. When both are high, we observed poor coverage of assembly, as shown in the bolded rows.

**Table 1:** Comparison of coverage and entropy loss of BAC sequences^a^.

Clone (BAC no.)	SequenceLength(bp)	SequenceCoverage (%)	CoverageDeficiency (%)	H^30^	I^30^	ΔH^30^	% of repeat
AC011809	108,767	99.98	0.02	5.335862	5.337411	0.03%	0.46%
AC002328	109,171	99.61	0.39	5.335947	5.339022	0.06%	0.99%
AC064879	109,180	99.68	0.32	5.335971	5.339058	0.06%	0.97%
AC023673	109,367	99.91	0.09	5.338616	5.339801	0.02%	0.38%
AC011713	109,694	98.85	1.15	5.332009	5.341098	0.17%	2.91%
AC009243	110,565	100.00	0.00	5.344373	5.344534	0.00%	0.02%
AC022520	110,611	99.63	0.37	5.341812	5.344714	0.05%	0.92%
**AC018460**	**110,619**	**66.48**	**33.52**	**4.99782**	**5.344746**	**6.49%**	**41.15%**
AC007764	111,222	99.84	0.16	5.346664	5.347107	0.01%	0.13%
AC000348	111,566	98.78	1.22	5.339937	5.348449	0.16%	2.81%
AC092191	80919	99.52	0.48	5.205896	5.208925	0.06%	1.01%
AC185533	95808	98.46	1.54	5.271617	5.2823	0.20%	3.41%
**AC018485**	**99441**	**82.86**	**17.14**	**5.094117**	**5.298469**	**3.86%**	**19.86%**
AC018478	103809	99.90	0.10	5.315052	5.317144	0.04%	0.56%
AC092242	111023	100.00	0.00	5.346276	5.346329	0.00%	0.01%
**AC018482**	**113821**	**87.42**	**12.48**	**5.261927**	**5.357142**	**1.78%**	**19.27%**
AC185534	119461	99.42	0.58	5.37266	5.378151	0.10%	1.75%
AC092399	122013	99.92	0.08	5.386815	5.387333	0.01%	0.17%
AC007837	123647	99.90	0.10	5.391958	5.393112	0.02%	0.28%
AC007329	126140	99.99	0.01	5.401638	5.401783	0.00%	0.05%

Sequence coverage percentages as listed in Dohm *et al* [10].

a The programs for the computation are available at: http://sourceforge.net/projects/seqentropy/files/SeqEntropy-demo-20130203.zip.

b The columns H30, I30,ΔH30 are computed by our program “SeqReadEntropy” using read length of 30 bp.

c The column “% of repeat” is computed by our program “SeqReadRepeat” using read length of 30 bp.

### Evaluation of the Limitation of Short-read Sequencing for Animal Genomes

We applied the entropy measurement to analyze the limitation of short-read sequencing for different organisms. We selected five model animals (Table 2) with genome sizes ranging from 10^7^∼10^9 ^bp. In order to calculate the entropy scores for large eukaryotic genomes on a desktop computer (Intel i7-3820 CPU and 8G RAM), we applied the principle behind Equation 8 above and divided up the input sequences into 256 (4^4^) or 1024 (4^5^) subsets based on the prefix (4mer or 5mer, respectively) of the sequences. The subsets from each genome are than run sequentially on the desktop computer. We had to run the processes sequentially due to memory constraint (only 8 Gb of RAM was available on the desktop computer) The total run-time for each organism is reported in Table 2. While large genomes such as that of human took a long time (295 hours) to complete, with some modifications to the program, we can run each subset in parallel on a computing cluster to reduce the run time significantly.

**Table 2:** Five animal genomes for entropy measurement.

Organism	Genome size	Version^a^	Computation time^b^
Yeast (*S. cerevisiae*)	1.2×10^7^	sacCer3	1.3 minutes
Nematode (*C. elegans*)	1.0×10^8^	ce10	33 minutes
Fruit fly (*D. melanogaster*)	1.3×10^8^	dmel_r5.42	42 minutes
Zebrafish (*D. rerio*)	1.4×10^9^	danRer7	66 hours
Human (*H. sapiens*)	3.2×10^9^	hg19, GRCh37.p5	295 hours

a The whole genome sequences were downloaded from http://hgdownload.cse.ucsc.edu/for the organisms: *S. cerevisiae*, *C. elegans*, *D. rerio*, and *H. sapiens* and ftp://ftp.flybase.net/for *D. melanogaster*.

b The computation time of entropy measurement was recorded for read length 100 bp on a PC with Intel i7-3820 CPU and 8G RAM.

**Figure 2:**
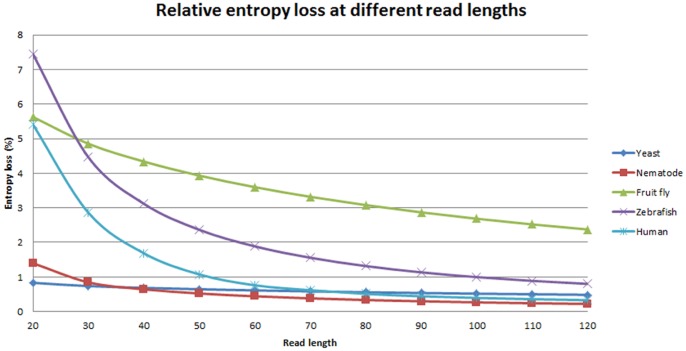
Entropy losses at different read lengths for different **organisms.** In the five organisms, the genomes of zebra fish (*D. rerio*) and fruit fly (*D. melanogaster*) will lose more entropy regardless of any read length used for sequencing. In particular, the fruit fly loses >2% of entropy loss even with read length of 120 bp. It will be <1% of entropy loss at read length of 230 bp. On the other hand, the genomes of Yeast (*S. cerevisiae*) and Nematode (*C. elegans*) have minor entropy loss even with very short reads. The detail results of entropy measurements are listed in **Table 5**.

Figure 2 depicts the relative entropy losses at different read lengths if idealized sequences are used for the organisms. Human requires read length of 60 bp and zebra fish requires read length of 100 bp to overcome the 1% entropy loss threshold. The genome size of zebra fish is less than half of human genome. It indicates that zebra fish genome is more repetitive than human genome. Moreover, the nematode, *C. elegans*, requires very short read (less than 30 bp) to avoid 1% entropy loss whereas fruit fly (*D. melanogaster*) requires more than 320 bp. Our analysis shows that genome assembly of many other organisms using short reads may be more challenging than human genome assembly.

The relationship between entropy loss and read length explains the limitation of short-read sequencing technology illustrated by Alkan *et al.* [4]. In the early experiments of the 1000 Genomes Project (like SRA ID: ERX000020), the read length of sequencing data is 36 bp and the curve of relative entropy loss for human genome in Figure 2 indicates more than 2% entropy loss at the read length of 36 bp. As a result, it is almost impossible to retrieve a perfect whole genome assembly from those WGS experiments. On the other hand, Sundquist *et al.* showed the sequencing of *D. melanogaster* still achieved worse genome sequence coverage than that of human chromosomes at read lengths of 200 bp [11]. Our proposed quantitative model illustrates the deficiency of sequence coverage for *D. melanogaster* comes from the richness of repeats in its genome.

### Evaluation of Bacterial Whole Genome Sequencing at Different Read Lengths

The *Escherichia coli* strain MG1655 whole genome shotgun sequencing datasets SRX000429 and SRX000430 generated using Illumina Genome Analyzer are commonly used as performance benchmark of short read sequencing [12]. The complete genome sequence of the same strain (NCBI REFSEQ ID: NC_000913) had been well characterized since 1997 [13]. Therefore, we can compare the result of the de novo assembly using the Illumina reads to the completed reference genome by mapping the contigs to the reference genome sequence.

To explore the reliability of the de novo assembly result, we computed the entropy at read length of 36 bp for the *E. coli* genome sequence NC_000913. We listed the entropy loss of the *E. coli* genome sequence along with some other prokaryotic whole genome sequences in Table 3. It shows that the entropy loss for the *E. coli* genome sequenced at read length of 36 bp is 0.22%. Compared this with the results obtained in Table 1, a relative entropy loss of 0.22% corresponds to about 2% genome coverage loss and suggests the difficulty in achieving a perfect genome coverage. Most of the assembly results without pair information by publicly available de novo assemblers can only achieve a genome-wide coverage of around 98% for the *E. coli* short reads dataset SRX000429 (https://wiki.nbic.nl/index.php/Raw_results_of_NGS_de_novo_assembly). With the help of longer reads or paired-end reads information available since the two 36 bp *E. coli* datasets were generated, the de novo assembly can achieve a better genome coverage than 98%. To approximate the effects of longer reads and paired-end reads, we calculate the relative entropy losses at k-substring length of 500 bp and 1000 bp (Table 3). However, based on the entropy losses, we think it is still very difficult to develop an automated de novo assembler to reach lossless assembly using data generated from current sequencing platforms. Manual inspections and the use of long range mapping information are necessary in most of the genome assembly. In general, our analysis indicates that smaller prokaryotic genomes with fewer repeats have less entropy loss compared to larger genomes. However, it’s the repeat structure and not the genome size that plays a determining role in entropy loss. *Bacteroides thetaiotaomicron* VPI-5482 has a larger genome but much fewer long repeats than *E. coli.* [14]. As a consequence, *B. thetaiotaomicron*’s entropy loss at read length 36 bps is more similar to that of a much smaller prokaryotic genome. Using entropy losses calculated at various read lengths, we can capture the repeat structure of a genome.

**Table 3:** Entropy loss of selected prokaryotic whole genomes with reads of lengths 36, 500 and 1000 bps.

Seq. no	Organism	SequenceLength(bp)	ΔH^36^	ΔH^500^	ΔH^1000^
NC_000913	E. *coli* K-12	4,639,675	0.22%	0.09%	0.04%
NC_004663	B. *thet* VPI-5482	6,260,361	0.15%	0.09%	0.05%
NC_008525	P.*pent* ATCC25745	1,832,387	0.16%	0.11%	0.08%
NC_000908	M.*geni* G37	580,076	0.11%	0.00%	0.00%

The third generation sequencing platforms use single-molecule technologies and other nanotechnologies [15]. The new methods claim to produce longer reads (>3000 bp). For instance, the reads for sequencing the *E. coli* genome could reach an average length of more than 3000 bps [16]. We showed the entropy loss at read length of 500 and 1000 bps for a few prokaryotic whole genome sequences. At the read length of 500 bps, *Mycoplasma genitalium*, an obligate parasitic bacterium with a highly reduced genome, has an insignificant entropy loss (Table 3).

**Figure 3:**
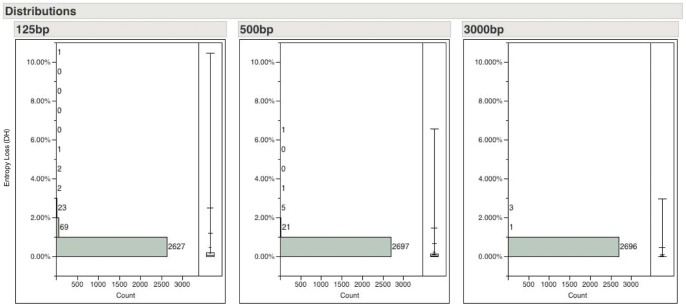
Histograms and quartile box plot of relative entropy losses in 2725 prokaryotic replicons. The x-axis shows the number of replicons in each bin while the y-axis shows the % entropy loss (ΔH). The quartile box plot displays the mean (diamond shape), the medium (50%) the first (25%) and the third (75%) quartiles (the boxes), and the entire range (the whiskers). The vast majority of the replicons lost <1% entropy regardless of the read length.

**Figure 4:**
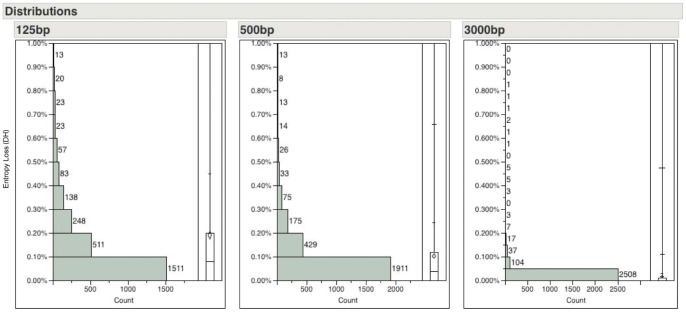
Histograms and quartile box plot of entropy losses in 2725 prokaryotic replicons truncated at 1% entropy loss in order to see the finer breakdown. The x-axis shows the number of replicons in each bin while the y-axis shows the % entropy loss (ΔH). The quartile box plot displays the mean (diamond shape), the medium (50%) the first (25%) and the third (75%) quartiles (the boxes), and the entire range (the whiskers). It is clear that as read length increases, the entropy loss decreases. As a result, a higher number of replicons have ΔH <1.0%.

In light of the new and emerging sequencing technologies, we applied the entropy calculation to 2725 prokaryotic chromosomes and plasmids which were downloaded from the NCBI FTP site (ftp://ftp.ncbi.nih.gov/genomes/Bacteria/- downloaded June 2012). We computed the relative entropy losses at read lengths of 125 bp, 500 bp and 3000 bp – roughly correspond to the read lengths of Illumina, Roche 454 pyrosequencing and PacBio SMRT platforms, respectively. We showed the distribution of the relative entropy loss scores in Figure 3. While there are some replicons, especially certain highly repetitive plasmids, with entropy loss scores >1.0% (see Table S1), the vast majority (>99%) of the prokaryotic replicons would lose less than 1.0% entropy with current sequencing technologies. These results are confirmed by the fact that the vast majority of the prokaryotic genomes can be reasonably reconstructed into long contigs with current shotgun sequencing technologies. In figure 4, we zoomed in to show only the results from replicons with entropy loss <1% in order to further differentiate the effect of read length. The entropy loss scores decrease as the read length increases. At read length of 125 bp, even if the sequencing reads cover the genomes evenly and completely, de novo assemblers without utilizing pair-end information would still not be able to fully reconstruct the genome due to the presence of repeats in ∼80% of the cases (2140/2725 with ΔH^125^>0). On the other hand, with read length of 3000 bp, 58% of the replicons surveyed could be reconstructed completely (i.e. no loss of entropy due to repeats). We list the top 10 genomes (excluding plasmids) with the largest entropy losses at read length of 125 bp, 500 bp and 3000 bp in **Table 4** and the complete results of the entropy loss scores are listed in **Table S1**. There are 41 chromosomes with ΔH^125^>1% and 6 chromosomes with ΔH^500^>1%. Many of these outliers have been noted in the literature to have highly repetitive genomes due to various evolutionary forces [17], [18], [19], [20].

**Table 4:** Prokaryotic chromosomes with largest entropy losses at read lengths of 125, 500 and 3000 bp.

Genome ID	ΔH^125^	Genome ID	ΔH^500^	Genome ID	ΔH^3000^
Bordetella pertussis Tohama I	1.79136%	Bordetella pertussis Tohama I	0.898868%	Mycoplasma agalactiae	0.367427%
Xanthomonas oryzae pv. oryzae PXO99A	1.82280%	Mycoplasma fermentans M64 chromosome	0.934389%	Dehalococcoides ethenogenes195	0.394745%
Wolbachia sp. wRi	1.98404%	Acinetobacter baumannii SDF	0.934389%	Mycoplasma fermentans M64	0.415779%
Aliivibrio salmonicida LFI1238chrom 1	2.04042%	Mycoplasma mycoides subsp. mycoides SC str. PG1	0.940714%	Orientia tsutsugamushi Boryong	0.416469%
Shigella boydii CDC 3083-94	2.09366%	Wolbachia endosymbiont ofCulex quinquefasciatus Pel	1.028366%	Methylobacillus flagellatus KT	0.42687%
Shigella dysenteriae Sd197	2.40508%	Aliivibrio salmonicida LFI1238chrom 1	1.153796%	Wolbachia sp. wRi	0.43966%
Acinetobacter baumannii SDF	2.64465%	Wolbachia sp. wRi	1.285340%	Alteromonas macleodi	0.465815%
Orientia tsutsugamushi str. Iked	2.67584%	Aliivibrio salmonicida LFI1238chrom 2	1.334486%	Bartonella tribocorum CIP105476	0.484289%
Mycoplasma mycoides subsp.mycoides SC str. PG1	2.75548%	Orientia tsutsugamushi str. Ikeda	1.570541%	Streptococcus agalactiaeNEM316	0.49117%
Orientia tsutsugamushi Boryong	4.62902%	Orientia tsutsugamushi Boryong	2.753110%	Candidatus Phytoplasma mali	0.693043%

a The complete entropy computations of 2725 prokaryotic replicons are listed in **Table S1**.

**Table 5:** The relative entropy losses of five animal genomes at different read lengths.

ReadLen k	ΔH^k^ of Yeast	ΔH^k^ of Nematode	ΔH^k^ of Fruit fly	ΔH^k^ of Zebrafish	ΔH^k^ of Human
20	**0.831217%**	1.397796%	5.627856%	7.44469%	5.406936%
30	0.736196%	**0.844718%**	4.852753%	4.461654%	2.858026%
40	0.683882%	0.642178%	4.337883%	3.11601%	1.684438%
50	0.644228%	0.522634%	3.932514%	2.363446%	1.072922%
60	0.611417%	0.440682%	3.599801%	1.886473%	**0.655126%**
70	0.582789%	0.380003%	3.319438%	1.556083%	0.482622%
80	0.557695%	0.332944%	3.078671%	1.315029%	0.379953%
90	0.536264%	0.295774%	2.868949%	1.134327%	0.313174%
100	0.517069%	0.265546%	2.684404%	**0.996147%**	0.2661318%
110	0.499602%	0.240705%	2.520669%	0.887384%	0.2306789%
120	0.483514%	0.219947%	2.374214%	0.799316%	0.2028979%
320	_	_	1.007846%	_	_
330	_	_	**0.973252%**	_	_

a The bold numbers show the relative entropy loss values and the corresponding minimal read lengths at which the relative entropy losses are below 1% for different animal genomes.

Taken together, these observations mean the third generation sequencing technologies can theoretically provide complete genome sequences for most prokaryotic organisms if the error rate is controlled or corrected by other short read sequencing technologies. On the other hand, bigger eukaryotic genomes, which are likely to have more complex repeat structure, would benefit less from the longer reads produced by third generation sequencing technologies.

## Discussion

The evaluation of k-substring entropy shows that the genomes of different organisms may have distinct repeat structures that impose limitation on sequencing at a certain read length regardless of their genome sizes. Using the entropy measurement, we can estimate an ideal read length for a given genome sequencing project by trying to minimize the entropy loss. Kingsford *et al.* previously introduced a de Bruijn graph method to evaluate the influence of repeat structure on sequence assembly [21]. However, their analysis was only applied to prokaryotic genomes which are relatively small (most <10 megabases). For the analysis of large genomes such as the mammalian or plant genomes, which can be a gigabases long, a program requires huge amount of memory to record all distinct substrings in the genome. Our algorithm is designed to handle genomes at any scale. With eq. (8), we can separate the substrings into different subsets by their prefix of length *m* (*m* = 5 for human genome and m = 4 for zebra fish genome) and calculate the entropy measurement of each set individually. As a result, the proposed program can be run on a desktop computer (8 Gb of RAM) for genomes of an arbitrary size at the cost of time. If completely parallelized, the calculation can be done on a large cluster to reduce the run time significantly. For a typical prokaryotic genome, it takes a few seconds to a few minutes to calculate the entropy loss at a given read length. The processing time is read length dependent and for read length <1000 bp, the calculation takes <1 min using a single CPU core. This length should be sufficient for most of the sequencing technologies in the near future with a few exceptions (e.g. Pacific Biosciences SMRT platform). For read length of 3000 bp, it takes about 20 min to calculate the entropy for one bacterial genome.

This task of entropy measurement can be performed on a preliminary assembly or on a reference genome from a closely related organism. Since read length depends on the sequencing platforms and the protocols used, it is often not possible to alter. Experimentally, it is easier to construct paired-end libraries with different insert sizes. We can use pair-end libraries of different insert sizes to mimic the effect of longer reads. Wetzel *et al.* recently demonstrated that the assembly outcome can be improved drastically by tuning mate-pair sizes (i.e. adjusting the average insert size of the pair-end library) to match the dominant repeat types [22]. Furthermore, they showed that “short” inserts that are between 4 to 6 times the actual read lengths perform better than long inserts that are a few kilobases long. This is because short inserts that barely span the repeats are more effective at resolving local ambiguities than long inserts. Their work is based on idealized de Bruijn graph reconstruction of a genome (see Kingsford *et al* 2010 for the method). This process is computationally and memory intensive. As a result, it is not easily scaled up to handle large eukaryotic genomes. On the contrary, our method can estimate the ideal read length and is fast and highly scalable as we have demonstrated in the previous sections.

We propose that SeqEntropy can be run with different read length parameters to detect the minimum entropy loss. Based on the work of Wetzel *et al*, we propose that the mate-pair sizes of the sequence library be slightly longer than the theoretical ideal read length detected by SeqEntropy. While the insert size is tuned, the actual read length is still based on available funding and sequencing platforms, allowing minimal interruption to existing and on-going sequencing projects. As more genomes are being sequenced in an automated fashion, the ability to tune the sequencing parameters to achieve better assemblies is highly desirable. We propose that entropy loss can be used to provide an accurate and objective estimate for the optimal sequence length.

### Availability

The source programs of this work are available from the Web site http://sourceforge.net/projects/seqentropy/.

## Supporting Information

Table S1 **List of 2725 Prokaryotic chromosomes.** The detail computations of entropy losses at read lengths of 125, 500 and 3000 bp for all 2725 Prokaryotic chromosomes.
(XLS)
